# Influence of cheek support on respiratory impedance measured by forced oscillation technique

**DOI:** 10.1186/2193-1801-2-342

**Published:** 2013-07-25

**Authors:** Akemi Uchida, Satoru Ito, Béla Suki, Hiroki Matsubara, Yoshinori Hasegawa

**Affiliations:** Department of Clinical Laboratory, Nagoya University Hospital, Nagoya, 466-8550 Japan; Department of Respiratory Medicine, Nagoya University School of Medicine, Nagoya, 466-8550 Japan; Department of Biomedical Engineering, Boston University, Boston, MA 02215 USA

**Keywords:** Asthma, Cheek support, COPD, Forced oscillation technique, MostGraph, Respiratory resistance, Reactance, Upper airway shunt

## Abstract

The forced oscillation technique (FOT) is a useful tool to assess respiratory resistance and reactance during tidal breathing in patients with respiratory diseases, specifically asthma and chronic obstructive pulmonary disease. Although the FOT has been clinically used, results of respiratory impedance can be affected by various factors such as upper airway artifact. We investigated the effects of cheek support on respiratory resistance and reactance measured by a commercially available FOT equipment MostGraph-01. Respiratory resistance at 20 Hz (R20) with support of the cheeks was significantly higher than those without the cheek support in healthy subjects. Two different cheek support protocols, support of the cheeks by subjects themselves and an operator, were compared in healthy volunteers and patients with respiratory diseases. The cheek support protocols significantly affected respiratory resistance at 5 Hz (R5) and reactance at 5 Hz (X5) in the patient group but not in the healthy subjects. Moreover, for X5, there was a significant interaction between cheek support protocols (by a subject or operator) and groups (healthy or diseased). In conclusion, during impedance measurements using the FOT, application of cheek support either by subjects or the operator is recommended to reduce upper airway artifacts, however, results obtained by two protocols may be different in patients with respiratory diseases. Contribution of the chest wall and position of the arms to the mechanical properties should be carefully considered in physiological studies in which the FOT is attempted.

## Background

The forced oscillation technique (FOT), as first described by Dubois et al. (1956), is an accurate and powerful method to assess the respiratory resistance (Rrs) and reactance (Xrs) from input impedance measurement made over a range of frequencies (Grimby et al. 1968;Michaelson et al. 1975). Using the FOT, the mechanical properties of the lung have been characterized in humans (Dubois et al. 1956;Kaczka et al. 1997). As commercially available instruments were developed, the FOT has been used to assess respiratory functions of patients with respiratory diseases such as asthma, chronic obstructive pulmonary disease (COPD), and interstitial lung disease (Yaegashi et al. 2007;Kanda et al. 2010;Paredi et al. 2010;Crim et al. 2011;Mori et al. 2011;Ohishi et al. 2011;Ito et al. 2012;Mori et al. 2013).

Although the commercially available FOT devices are useful, the results of respiratory mechanics can be affected by various factors and artifacts. To interpret data obtained by the FOT properly, it is necessary to address potential artifacts that might otherwise be misleading (Oostveen et al. 2003;Goldman et al. 2005). Because pressure oscillations are applied at the mouth, the impedance of extra-thoracic airway walls, including cheeks, tongue, mouthpiece and upper airways, affects the results of the measurements (Oostveen et al. 2003;Goldman et al. 2005). The reason is that a component of the measured input flow can be lost in the oscillatory motion of the compliant upper airway walls and is unable to enter the lower respiratory system properly (Peslin et al. 1985a;Cauberghs and Van De Woestijne 1989;Farre et al. 1999). In order to eliminate the upper airway artifacts, the support of the cheeks with the palms of both hands either by the subject or an operator is recommended (Oostveen et al. 2003). Technicians need to support the cheeks of patients when the patients have difficulty in supporting their cheeks firmly by themselves. However, it is not known whether the respiratory impedance spectra obtained with the cheeks supported by the subject are same as those supported by an operator.

The purpose of the present study was to carefully characterize the effects of cheek support on respiratory impedance measurements. Two different cheek support protocols, support of the cheeks by subjects themselves and by an operator, were compared in healthy subjects and patients with respiratory diseases. Impedance data collection was made using a commercially available FOT machine (MostGraph-01; Chest M.I., Tokyo, Japan) (Mori et al. 2011;Yamauchi et al. 2012).

## Results

### Characteristics of healthy subjects and patients with respiratory diseases

The characteristics and pulmonary function test results of healthy subjects (n = 10) and the patients with respiratory diseases (n = 18) are shown in Table 1. Respiratory diseases involve asthma (n = 7), COPD (n = 4), and interstitial lung diseases (n = 7). There were statistically significant differences in age (P < 0.001), forced vital capacity (FVC) (P = 0.006), forced expiratory volume in one second (FEV_1_) (P < 0.001), FEV_1_/FVC ratio (P = 0.005) between healthy subjects and patients (Table 1).

**Table 1:** **Clinical characteristics of investigated subjects**

	Healthy (n = 10)	Disease (n = 18)
		Asthma (7), ILD (7), COPD (4)
Age, years (range)	38.4 ± 9.6 (24–59)	67.9 ±9.9* (51–82)
Sex, male/female	4/6	11/7
Height, cm	164.0 ± 9.3	158.8 ± 8.6
Weight, kg	59.8 ± 10.2	60.1 ± 9.7
BMI	22.1 ± 3.5	23.9 ± 12.7
Pulmonary function test results		
FVC, L	3.99 ± 1.08	2.86 ± 0.88*
FEV_1_, L	3.27 ± 0.78	2.02 ± 0.61*
FEV_1_, % predicted	107.1 ± 16.5	95.8 ± 18.0
FEV_1_/FVC, %	82.5 ± 5.8	71.2 ± 10.7*

Values are mean ± SD. *: Significantly different (P < 0.05) vs. healthy subjects (t-test).

*COPD* chronic obstructive pulmonary disease, *ILD* interstitial lung disease, *BMI* body mass index, *FVC* forced vital capacity, *FEV*_*1*_ forced expiratory volume in 1 s.

### Effects of cheek support on respiratory mechanics in healthy subjects

**Figure 1:**
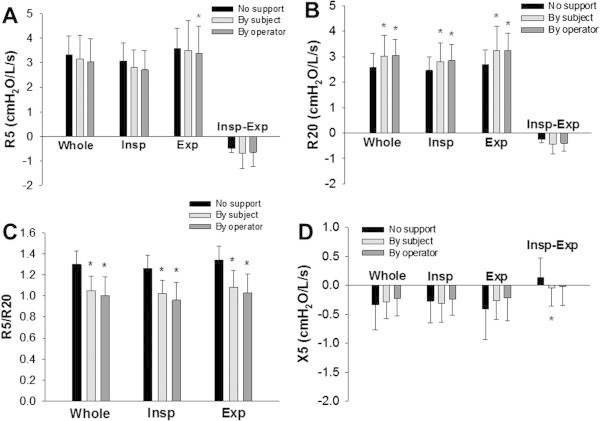
**Effects of cheek support on respiratory mechanics in healthy subjects.** The respiratory impedance was measured without cheek support and with cheeks supported by a subject and an operator. Rrs at 5 Hz (R5; **A**) and at 20 Hz (R20; **B**), R5/R20 **(C)**, and Xrs at 5 Hz (X5; **D**) during a whole-breath (Whole), inspiratory (Insp) and expiratory (Exp) phases, and the difference (Δ) between inspiratory and expiratory phases (Insp-Exp) are shown. Values are mean ± SD. *: Significantly different (P < 0.05) vs. without cheek support by one-way repeated measure ANOVA followed by Bonferroni test for post hoc analysis (n = 10).

Effects of cheek support on respiratory impedance measured by the FOT were examined in healthy subjects (n = 10). First, impedance was measured without cheek support, and then followed by measurements during which the cheeks were supported by subjects. Results of the respiratory mechanics are shown in Figure 1. Rrs at 5 Hz (R5) and 20 Hz (R20) and Xrs at 5 Hz (X5) were analyzed. Each parameter was expressed as mean values during a respiratory cycle (whole-breath), inspiratory and expiratory phases, and the differences between inspiratory and expiratory phases (Δ) according to previous reports by other laboratories (Dellaca et al. 2004;Paredi et al. 2010;Mori et al. 2011;Ohishi et al. 2011). Furthermore, to examine the involvement of frequency-dependent changes in Rrs (Grimby et al. 1968), R5/R20 ratios of whole-breath, inspiratory and expiratory phases were also compared. Although the difference between R5 and R20 (R5 - R20) derived from consumer product with normal setting has widely been used as an index of frequency-dependence (Kanda et al. 2010;Mori et al. 2013), the R5/R20 ratio was used in this study instead of R5 - R20 in order to minimize the influence of the absolute values of R5 or R20 on the index of heterogeneity.

There was no significant difference in R5 (Figure 1A). The values of R20 for whole-breath, inspiratory and expiratory phases with cheek support were significantly higher than those without cheek support (P < 0.05) (Figure 1B). Values of R5/R20 for whole-breath, inspiratory, and expiratory phases with cheek support by subjects were significantly lower than those without cheek support (P < 0.001) (Figure 1C). Moreover, the values of the difference between inspiratory and expiratory phases in X5 (ΔX5) were slightly but significantly affected by application of cheek support by the subjects (P = 0.011) (Figure 1D). There was no significant difference in X5 for whole-breath, inspiratory or expiratory phases between the groups (Figure 1D).

### Comparison of cheek support by a subject and an operator in healthy subjects

**Figure 2:**
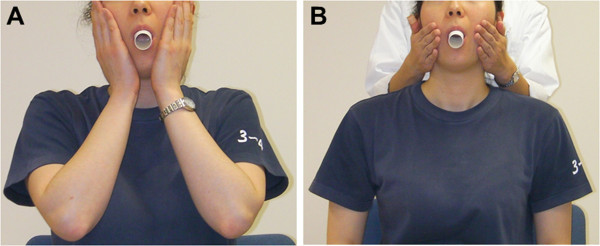
**Examples of cheek support by a subject and an operator.** During the impedance measurements, cheeks were supported by a subjects **(A)** and an operator standing behind the subjects **(B)**.

We examined whether the respiratory impedance spectra with the cheeks supported by the subject are different from those supported by the operator. For consistency, the same operator (A.U., a technician) supported the cheeks of each subject throughout the study. First, the cheeks were supported by the subjects themselves during the impedance measurements (Figure 2A). Next, the operator stood behind the subjects and supported the cheeks by her palms (Figure 2B).

In healthy subjects (n = 10), the results of respiratory impedance with support of the cheeks by the subjects were not significantly different from those supported by the operator (Figure 1A-D). R5 during expiratory phase with cheek support by an operator was slightly but significantly lower than that without cheek support (Figure 1A).

### Comparison of cheek support by a subject and an operator in patients with respiratory diseases

**Figure 3:**
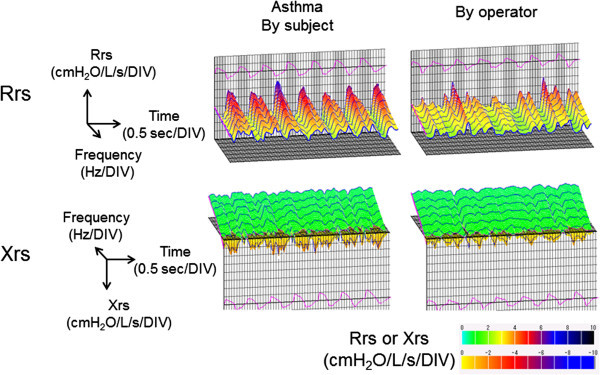
**Representative examples of colored 3-dimensional images of Rrs and Xrs with asthma cheeks support by a subject and an operator in a patient with asthma are shown.**

**Figure 4:**
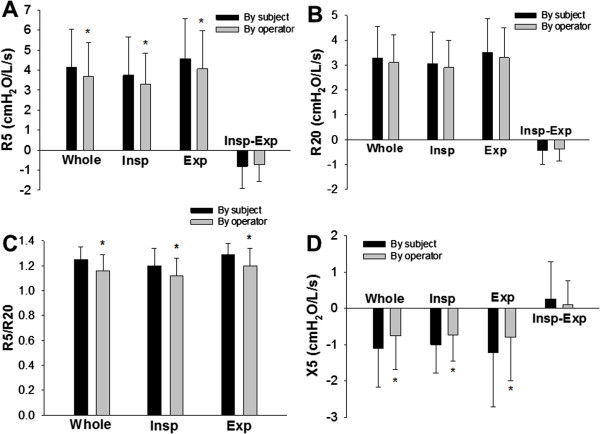
**Effects of cheek support on respiratory mechanics in patients with respiratory diseases.** The respiratory impedance was measured with cheeks supported by a subject and an operator. Rrs at 5 Hz (R5; **A**) and at 20 Hz (R20; **B**), R5/R20 **(C)**, and Xrs at 5 Hz (X5; **D**) during a whole-breath (Whole), inspiratory (Insp) and expiratory (Exp) phases, and the difference (Δ) between inspiratory and expiratory phases (Insp-Exp) are shown. Values are mean ± SD. *: Significantly different (P < 0.05) vs. cheek support by subjects by paired t-test (n = 18).

Representative examples of colored 3-dimensional images of Rrs and Xrs of a patient with asthma with cheek support by the subject and the operator are shown in Figure 3. In the patient group (n = 18), the values of R5, whole-breath, inspiratory and expiratory phases, with cheek supported by subjects were significantly higher than those by an operator (Figure 4A). In contrast, there was no significant difference in the results of R20 or ΔR5 (Figure 4A and B). As a result, R5/R20 ratios for whole-breath, inspiratory and expiratory phases with support of the cheeks by the subjects were significantly higher than those supported by the operator (Figure 4C). The values of X5 for whole-breath, inspiratory and expiratory phases with the cheeks supported by the subjects were significantly lower (more negative) than those supported by the operator (Figure 4D).

The differences in the values of R5, R20, or X5 between two cheek support protocols during whole breath of the diseased group were 0.457 ± 0.593, 0.178 ± 0.441, 0.312 ± 0.342 cmH_2_O/L/s, respectively (n = 18). These differences in the R5, R20, or X5 did not significantly depend on the baseline values (R5, R20 or X5), sex (male/female), age, disease (asthma, COPD, or interstitial lung disease), % predicted FEV_1_, or FEV_1_/FVC.

### Significant effects of cheek support protocols on respiratory impedance of patients

We further examined whether the cheek support protocols statistically affected respiratory impedance spectra of healthy subjects (n = 10) and patients with respiratory diseases (n = 18) using two-way repeated measure analysis of variance (ANOVA) (Table 2). R5, R5/R20 ratios, and X5 during whole breath, inspiratory and expiratory phases were significantly dependent on cheek support protocols (Table 2). R5/R20 ratios were significantly higher and X5 during whole breath and inspiratory phases were significantly lower (more negative) in the diseased group than in the healthy group (Table 2). Moreover, there was a statistically significant interaction in X5 during whole breath (P = 0.005) and expiratory phase (P = 0.01) between the cheek support protocols and groups (healthy or diseased) (Table 2). These statistical results demonstrated that cheek support protocols differently affected X5 values between healthy and diseased groups. R20 values, ΔR5, or ΔX5 were not significantly affected by cheek support protocols.

**Table 2:** **Effects of cheek support protocols on respiratory impedance spectra in healthy and diseased groups**

Impedance parameters		Cheek support protocols	Healthy vs. diseased groups	Interaction between cheek support protocols and groups
R5, cmH_2_O/L/s	Whole	P = 0.007	NS	NS
	Inspiratory	P = 0.017	NS	NS
	Expiratory	P = 0.017	NS	NS
	Δ	NS	NS	NS
R20, cmH_2_O/L/s	Whole	NS	NS	NS
	Inspiratory	NS	NS	NS
	Expiratory	NS	NS	NS
	Δ	NS	NS	NS
R5/R20	Whole	P = 0.001	P < 0.001	NS
	Inspiratory	P < 0.001	P = 0.003	NS
	Expiratory	P = 0.005	P < 0.001	NS
X5, cmH_2_O/L/s	Whole	P < 0.001	P = 0.044	P = 0.005
	Inspiratory	P = 0.003	P = 0.024	NS
	Expiratory	P = 0.002	NS	P = 0.01
	Δ	NS	NS	NS

Difference between two cheek support protocols (supported by subjects and the operator) in respiratory impedance during a whole-breath (Whole), inspiratory and expiratory phases, and the difference (Δ) between inspiratory and expiratory phases were compared by two-way repeated measure ANOVA followed by Bonferroni test for post hoc analysis in healthy subjects (n = 10) and patients with respiratory diseases (n = 18).

### Effects of cheek support protocols in healthy subjects and patients with asthma or interstitial lung disease

Since the number of COPD patients was small (n = 4), we next examined effects of the cheek support protocols on respiratory impedance spectra of healthy subjects (n = 10), asthma (n = 7), and interstitial lung disease (n = 7) except COPD. Similar to the results including COPD data, R5, R5/R20 ratios, and X5 during whole breath, inspiratory and expiratory phases were significantly dependent on cheek support protocol by two-way repeated measure ANOVA (Table 3). R5 during whole breath, R5/R20 ratios, and X5 during whole breath and inspiratory phase were significantly different between the cheek support protocols in patients with interstitial lung disease (Table 3). In patients with asthma, only X5 during whole breath was significantly dependent on cheek support protocol (Table 3). On the other hand, cheek support protocol did not significantly affect impedance results in healthy subjects. R5/R20 ratios were significantly higher and X5 during whole breath and inspiratory phases were significantly lower (more negative) in the asthma group than in the healthy group (Table 3). Moreover, there was a statistically significant interaction in R5 during whole breath and X5 during whole breath and inspiratory phase between the cheek support protocols and groups (Table 3). These statistical results demonstrated that cheek support protocols affected impedance results in patients specifically in those with interstitial lung disease.

**Table 3:** **Effects of cheek support protocols on respiratory impedance spectra except COPD group**

Impedance parameters		Cheek support protocols	Groups	Interaction between cheek support protocols and groups
			(healthy, asthma, ILD)	
R5, cmH_2_O/L/s	Whole	P = 0.002*	NS	P = 0.034
	Inspiratory	P = 0.007	NS	NS
	Expiratory	P = 0.009	NS	NS
	Δ	NS	NS	NS
R20, cmH_2_O/L/s	Whole	NS	NS	NS
	Inspiratory	NS	NS	NS
	Expiratory	NS	NS	NS
	Δ	NS	NS	NS
R5/R20	Whole	P = 0.003*	P = 0.003†	NS
	Inspiratory	P = 0.002*	P = 0.006†	NS
	Expiratory	P = 0.009*	P = 0.002†	NS
X5, cmH_2_O/L/s	Whole	P < 0.001*, **	P = 0.040†	P = 0.033
	Inspiratory	P < 0.001*	P = 0.032†	P = 0.044
	Expiratory	P = 0.002	NS	NS
	Δ	NS	NS	NS

Difference between two cheek support protocols (supported by subjects and the operator) in respiratory impedance during a whole-breath (Whole), inspiratory and expiratory phases, and the difference (Δ) between inspiratory and expiratory phases were compared by two-way repeated measure ANOVA followed by Bonferroni test for post hoc analysis in healthy subjects (n = 10), patients with asthma (n = 7), and interstitial lung disease (ILD) (n = 7). Significantly different (P < 0.05) between cheek support protocols in ILD (*) or asthma (**) group. †: Significantly different (P < 0.05) between asthma and healthy groups.

## Discussion

The main findings of the present study are that 1) respiratory system resistance measured by the FOT was significantly influenced by the application of cheek support in the healthy subjects; 2) respiratory impedance spectra, specifically X5, with support of cheeks by the subjects were significantly different from those supported by the operator in patients with respiratory diseases but not in healthy subjects.

One important factor that affects the values of the mechanical parameters measured by the FOT is the upper airway artifacts (Cauberghs and Van De Woestijne 1983;Peslin et al. 1985b). The cheek support is useful and convenient to reduce the effects of upper airway artifacts during the FOT measurements as recommended by the guideline (Oostveen et al. 2003), although it cannot remove the artifacts perfectly (Peslin et al. 1985a;Peslin et al. 1985b). In the present study, support of the cheeks significantly affected respiratory impedance in healthy subjects (Figure 1), consistent with findings in previous reports (Peslin et al. 1985a;Cauberghs and Van De Woestijne 1989). Application of cheek support increased the values of Rrs parameters at higher frequency (20 Hz) during whole-breath, inspiratory and expiratory phases (Figure 1B) by reducing the oscillatory flow into the upper airway wall impedance. In contrast, at the lower frequency, the values of R5 or X5 during whole-breath, inspiratory and expiratory phases were not different between with and without the application of cheek support (Figure 1). Our results demonstrate that Rrs is underestimated because of the influence of mechanical properties of the cheeks at higher frequency. Goldman et al. (2002) reported that manual support of the cheeks by hands affected Rrs values above 15 Hz in asthmatic adolescents. Our data in healthy adults are consistent with their findings.

To our knowledge, this is the first study to compare two different protocols for cheek support, by the subjects themselves and an operator, during respiratory impedance measurements. Respiratory impedance measured with support of the cheek by the subjects was significantly different from those supported by the operator in patients with respiratory diseases. The values of R5 significantly decreased while the values of X5 significantly increased when the cheek was supported by the operator (Figure 4). On the other hand, cheek support protocols did not affect the results of respiratory impedance measurements in healthy subjects (Figure 1). Moreover, statistical results demonstrated that the effects of the cheek support protocols were significantly different between healthy and diseased groups in analyzing X5 values (Table 2). Additionally, impedance results were strongly affected by cheek support protocol specifically in patients with interstitial lung disease (Table 3). One possible reason for this difference between the two cheek support techniques in the patient group is effects of the position of arms and chest walls during measurements. It is known that the mechanical properties of the chest wall significantly contribute to respiratory mechanics (Nagels et al. 1980;Hirai et al. 1999). Goldman et al. (2002) described that in the sitting position, support of cheeks with the palms introduces a potential adverse mechanical loading effect on the chest. Therefore, to raise their arms during supporting their cheeks may have affected breathing, specifically in patients with respiratory diseases in our cohort. Moreover, the frequency-dependence of Rrs, which was expressed as the R5/R20 ratio, was significantly increased when the cheeks were supported by the subjects themselves in the patient group (Figure 4C) specifically in patients with interstitial lung disease (Table 3). These results may be explained by the difference in chest wall configuration (Sakai et al. 2001) or an increase in airway-related heterogeneities (Lutchen et al. 1996;Suki et al. 1997;Ito et al. 2007;Kaczka et al. 2011) as a consequence of altered chest wall configuration. Another possibility is that aging also contributes to the difference because the healthy controls in our study were much younger than the diseased group. However, the changes in Rrs or Xrs parameters between the two protocols for cheek support did not correlate with age in the patient group (from 51 to 82 years old) or the healthy group (from 24 to 59 years old). Furthermore, even when all of the data including both healthy and diseased subjects (n = 28) were analyzed, the changes in impedance parameters did not correlate with age except the changes in X5 for inspiratory (R = −0.43, P = 0.023) and expiratory phases (R = −0.39, P = 0.039). Thus, it is likely that effects of aging on impedance measurements by our cheek support protocols were relatively small.

One of the benefits of the FOT above the breathing frequency is that it enables to measure both inspiratory and expiratory parameters which in turn allows the detection of expiratory flow limitation (Dellaca et al. 2004). Previous studies have demonstrated that the Rrs and Xrs values in the inspiratory and expiratory phases are different specifically in patients with COPD (Cauberghs and Van De Woestijne 1992;Lutchen and Gillis 1997;Kanda et al. 2010;Ohishi et al. 2011). Indeed, Rrs parameters, R5 and R20, were significantly larger in the expiratory phase than in the inspiratory phase both in healthy subjects and patients with respiratory diseases (Figures 1 and 4). However, this difference during the respiratory cycle was not affected by cheek support protocols. In contrast, ΔX5 values with cheeks supported by the subject became significantly lower compared with those without cheek support in healthy subjects (Figure 1D). Although the difference is very small (0.130 vs. -0.051 in mean values of ΔX5), this is potentially important given that it is the reactance that detects flow limitation when it becomes smaller than a fixed threshold (Dellaca et al. 2004).

This study has several limitations. The data were retrospectively collected in patients with respiratory diseases including COPD, asthma, and interstitial lung diseases and sample size for each disease was small. The cheek support protocol was not randomized but administered in fixed order. As reported by other laboratories (Paredi et al. 2010;Mori et al. 2011;Ohishi et al. 2011), the support of the cheeks has been routinely performed by patients themselves in our institution. In a few cases of the present study, it was difficult for the patients to support their cheeks by themselves. Therefore, there could be more variability introduced in the cheek support by the subjects which might have affected the results. Nevertheless, cheek support protocols did not affect values of respiratory impedance in healthy subjects, suggesting that our results likely derive from existence of respiratory diseases. Prospective studies with larger number of subjects may be necessary to confirm this. Another potential limitation is the impulse forcing implemented in the device. An impulse has a wide distribution of energy whereas the evaluation occurs only at selected frequencies which can reduce the desired signal-to-noise ratio of the measurement compared to pseudorandom noise or monofrequency oscillations.

In summary, respiratory mechanical parameters of patients with respiratory diseases were different between two protocols for cheek support, both of which are recommended in the guidelines. In contrast, the respiratory impedance parameters were not affected by the cheek support protocols in normal subjects. Postures and conditions during tidal breathing should carefully be considered for characterization of impedance data measured by the FOT specifically in patients with respiratory diseases. Future studies should standardize cheek support techniques for clinical applications of the FOT.

## Methods

### Subjects

Patients with respiratory diseases (n = 18) who attended the outpatient clinics of Nagoya University Hospital for measurements of respiratory functions and impedance between July 2010 and November 2012 were enrolled in this study. Healthy subjects without respiratory diseases (n = 10) were recruited from the hospital staff in order to standardize the impedance measurements in the institution. The results were retrospectively analyzed. The retrospective study was approved by the local ethics committee of Nagoya University Hospital.

### Impedance measurements by forced oscillation technique

Impedance data collection was made by the FOT using a commercially available machine (MostGraph-01; Chest M.I., Tokyo, Japan) which generates a broad-band waveform containing energy every 4 Hz from 4 Hz to 36 Hz. Impulse oscillatory signals generated by a loud speaker at intervals of 0.25 s were applied to the respiratory system through a paper mouthpiece (Chest M.I.) with a bacterial filter (SpirofilterC-3F; Chest M.I.) during tidal breathing at rest. Mouth pressure and flow signals were measured and used to calculate respiratory impedance by the system computer algorithms based on the standard recommendation (Oostveen et al. 2003). Using MostGraph-01, colored 3-dimensional plots of Rrs and Xrs are visualized as shown in Figure 3 and originally introduced by Mori et al. (2011).

### Protocols for cheek support and measurement conditions

Impedance measurements were performed in the sitting position using a nose-clip with their neck in a comfortable neutral posture. The subject was instructed to breathe quietly at functional residual capacity (FRC) level. After stabilization, respiratory impedance was recorded for approximately 20 s. Impedance measurements were done in a fixed order as follows. First, impedance measurements were performed without cheek support in healthy subjects. Next, subjects supported their cheeks firmly by the palms. Then, an operator (A.U., a technician) supported the cheeks of the patients using both of her hands. In patients with respiratory diseases, impedance was not measured without cheek support. Position of tongue and mouthpiece during measurements was carefully checked by the operator.

### Data analysis for impedance measurements

Impedance data were reviewed after measurements to check segments of breathing affected by artifacts such as coughing or swallowing. A total of three to five technically acceptable measurements were performed as recommended in the guideline (Oostveen et al. 2003). Variability expressed as the coefficient of variation of obtained Rrs (R5 or R20) data from acceptable measurements was less than 10%, and the mean value of those data was used for statistical analysis.

### Measurements of pulmonary function tests

Spirometry and lung volumes were determined using computerized equipment (Fudak70, Fukuda, Tokyo, Japan) according to the recommendation of the American Thoracic Society (1995). Spirometry was performed after impedance measurements.

### Statistical analysis

All data were expressed as means ± S.D. Student's t-test and ANOVA followed by Bonferroni test for post hoc analysis were used to evaluate the significance of differences between means and variances, with P < 0.05 as the level of significance (SigmaPlot11.0, Systat Software, San Jose, CA, USA). The chi-square test was also used to evaluate significance in group differences in various categories. Correlations between valuables were analyzed using the Spearman’s rank or Pearson’s correlation coefficient.

## Abbreviations

COPD: Chronic obstructive pulmonary disease
Δ: The difference between inspiratory and expiratory phases
FEV1: Forced expiratory volume in one second
FOT: Forced oscillation technique
FRC: Functional residual capacity
FVC: Forced vital capacity
Rrs: Respiratory resistance
R5: Rrs at 5 Hz
R20: Rrs at 20 Hz
Xrs: Respiratory reactance
X5: Xrs at 5 Hz
